# Binimetinib inhibits MEK and is effective against neuroblastoma tumor cells with low NF1 expression

**DOI:** 10.1186/s12885-016-2199-z

**Published:** 2016-03-01

**Authors:** Sarah E. Woodfield, Linna Zhang, Kathleen A. Scorsone, Yin Liu, Peter E. Zage

**Affiliations:** Department of Pediatrics, Section of Hematology-Oncology, Baylor College of Medicine, Houston, TX USA; Department of Neurobiology and Anatomy, The University of Texas Medical School, Houston, TX USA; Department of Biomedical Engineering, University of Texas at Austin, Austin, TX USA; Texas Children’s Cancer Center, Houston, TX USA

**Keywords:** Neuroblastoma, MEK162, Binimetinib, MAPK, MEK, NF1, ERK

## Abstract

**Background:**

Novel therapies are needed for children with high-risk and relapsed neuroblastoma. We hypothesized that MAPK/ERK kinase (MEK) inhibition with the novel MEK1/2 inhibitor binimetinib would be effective in neuroblastoma preclinical models.

**Methods:**

Levels of total and phosphorylated MEK and extracellular signal-regulated kinase (ERK) were examined in primary neuroblastoma tumor samples and in neuroblastoma cell lines by Western blot. A panel of established neuroblastoma tumor cell lines was treated with increasing concentrations of binimetinib, and their viability was determined using MTT assays. Western blot analyses were performed to examine changes in total and phosphorylated MEK and ERK and to measure apoptosis in neuroblastoma tumor cells after binimetinib treatment. NF1 protein levels in neuroblastoma cell lines were determined using Western blot assays. Gene expression of *NF1* and *MEK1* was examined in relationship to neuroblastoma patient outcomes.

**Results:**

Both primary neuroblastoma tumor samples and cell lines showed detectable levels of total and phosphorylated MEK and ERK. IC_50_ values for cells sensitive to binimetinib ranged from 8 nM to 1.16 μM, while resistant cells did not demonstrate any significant reduction in cell viability with doses exceeding 15 μM. Sensitive cells showed higher endogenous expression of phosphorylated MEK and ERK. Gene expression of *NF1*, but not *MEK1*, correlated with patient outcomes in neuroblastoma, and NF1 protein expression also correlated with responses to binimetinib.

**Conclusions:**

Neuroblastoma tumor cells show a range of sensitivities to the novel MEK inhibitor binimetinib. In response to binimetinib, sensitive cells demonstrated complete loss of phosphorylated ERK, while resistant cells demonstrated either incomplete loss of ERK phosphorylation or minimal effects on MEK phosphorylation, suggesting alternative mechanisms of resistance. NF1 protein expression correlated with responses to binimetinib, supporting the use of NF1 as a biomarker to identify patients that may respond to MEK inhibition. MEK inhibition therefore represents a potential new therapeutic strategy for neuroblastoma.

**Electronic supplementary material:**

The online version of this article (doi:10.1186/s12885-016-2199-z) contains supplementary material, which is available to authorized users.

## Background

Neuroblastoma is the most common extracranial solid tumor in children, and patients with high-risk disease have very poor outcomes, with long term disease-free survival rates between 35 and 45 % despite aggressive treatment regimens [1–3]. High-risk cases are characterized by frequent relapses and tumors resistant to established treatment, and novel therapies are sorely needed for patients with high-risk and relapsed neuroblastoma. Since aberrant growth factor receptor expression and activity have been shown to contribute to neuroblastoma pathogenesis, downstream intracellular signaling pathways, including the RAS/mitogen-activated protein kinase (MAPK) pathway, represent potential therapeutic targets.

The RAS/MAPK signaling pathway is one of the most frequently dysregulated signaling cascades in human cancer. In the canonical pathway, activity of the small GTPase RAS leads to sequential phosphorylation and activation of three protein kinases, BRAF, MAPK/extracellular signal-regulated kinase (ERK) kinase 1/2 (MEK1/2), and extracellular signal-regulated kinase 1/2 (ERK1/2) [4, 5]. Physiological activation of MEK1/2 and ERK1/2 is required for multiple normal cellular processes; however, overactivation of the pathway can lead to malignant transformation. Both MEK1 and MEK2 represent potential targets for therapeutic development due to their homology, narrow substrate specificities, and unique structural characteristics.

Targeting MEK1/2 to inhibit the oncogenic activity of the RAS/MAPK signaling pathway has been shown to be effective in *in vitro* and *in vivo* preclinical studies [6–11]. Inhibitor binding to the MEK1/2 proteins leads to conformational changes that lock unphosphorylated MEK1/2 into catalytically inactive states [12–14]. Since this inhibitor binding site is separate from the ATP-binding site, the mechanism of inhibition is independent of ATP and, thus, off-target effects are largely avoided [14, 15]. Such studies have led to the development of more than a dozen small-molecule inhibitors of MEK. Binimetinib is an ATP-noncompetitive inhibitor of both MEK1 and MEK2. Initial *in vitro* kinase assays demonstrated MEK inhibition with an IC_50_ of 12 nM without inhibition of other kinases at doses up to 10 μM [16, 17], and the safety and pharmacokinetics of binimetinib have been evaluated in adult cancer patients in multiple phase I and II studies [18–26].

The role of the RAS/MAPK pathway in neuroblastoma pathogenesis is poorly understood. Activating mutations in the genes of members of the RAS-MAPK pathway have been identified in a small subset of neuroblastoma tumors at diagnosis [27] and in many neuroblastoma tumors after relapse [28]. Furthermore, recent studies have identified a potential role for the Ras-GTPase activating protein (RasGAP) NF1 as a mediator of CRA resistance in neuroblastoma cells [29], suggesting key roles for the RAS/MAPK pathway both in neuroblastoma differentiation and relapse. Based on the evidence for a role of RAS/MAPK signaling in oncogenesis, we hypothesized that binimetinib may show significant antitumor activity in preclinical studies of neuroblastoma.

## Methods

### Cells and culture conditions

The neuroblastoma cell lines used in this study have been previously described [30–38] and were generously provided by Shahab Asgharzadeh (Children’s Hospital Los Angeles, Los Angeles, CA), Susan Cohn (The University of Chicago Children’s Hospital, Chicago, IL), Jill Lahti (St. Jude Children’s Research Hospital, Memphis, TN), John Maris (Children’s Hospital of Philadelphia, Philadelphia, PA), William Weiss (The University of California, San Francisco, San Francisco, CA) or were purchased from the American Type Culture Collection (ATCC; Rockville, MD). Cell lines were grown at 37° in 5 % CO_2_ in appropriate media (Invitrogen, Carlsbad, CA) supplemented with 10 % heat-inactivated fetal bovine serum (FBS) (Life Technologies, Grand Island, NY), L-glutamine, sodium pyruvate, and non-essential amino acids [39]. All cell lines were authenticated by deoxyribonucleic acid (DNA) profiling prior to use.

### Patient-derived tumor samples

The patient tumor samples employed in these studies were obtained from the Texas Children’s Hospital Research Tissue Support Services tissue bank. Fresh, resected neuroblastoma tumor samples were collected from patients after informed consent from either the patients or their guardians was obtained via an Institutional Review Board-approved tissue banking protocol. Samples were placed in sterile human stem cell media at the time of collection and flash frozen in liquid nitrogen for storage. All experiments on patient tissue samples were performed in compliance with the Helsinki Declaration and were approved by the Baylor College of Medicine Institutional Review Board (H-29553).

### Therapeutic agents

Binimetinib was generously provided by Novartis, Inc.. A 10 mM stock solution was generated in dimethyl sulfoxide (DMSO; Sigma-Aldrich, St. Louis, MO) and stored at −20 °C. Binimetinib was diluted in PBS or appropriate media immediately before use.

### RAS/MAPK assays

Patient tumor samples were homogenized and incubated for 30 min in radioimmunoprecipitation assay (RIPA) protein lysis buffer containing protease inhibitors (Sigma) and phosphatase inhibitors (Roche, San Francisco, CA) with homogenization every 10 min as previously described [39]. Lysates were centrifuged and supernatants were collected. Neuroblastoma cells were plated in 100-mm plates and allowed to adhere and proliferate for 48 h. Media was replaced 24 h after plating. Cells from plates at approximately 80 % confluency were then harvested and lysed as above.

To measure the effects of binimetinib on MEK and ERK phosphorylation, 2 × 10^6^ neuroblastoma cells were plated in 60-mm plates and allowed to adhere and proliferate for 48 h. Media was replaced 24 h after plating. Cells were treated with either 1 μM binimetinib or media alone (vehicle treatment) for one hour. Cells were harvested and lysed as above at the completion of each experiment.

Protein concentration in each sample lysate was measured using a protein assay dye reagent (Bio-Rad, Hercules, CA). 30–50 μg total denatured protein from each cell line or tumor sample lysate was separated by sodium dodecyl sulfate-polyacrylamide gel electrophoresis (SDS-PAGE) and transferred to nitrocellulose or polyvinylidene fluoride (PVDF) membranes (Invitrogen, Carlsbad, CA) using standard techniques. Membranes were blocked in Odyssey blocking buffer (Li-Cor, Lincoln, NE) for two hours at room temperature and then incubated overnight with primary antibodies to total MEK (9126; 1:1000; Cell Signaling, Danvers, MA), phosphorylated MEK (9154; 1:1000; Cell Signaling), total ERK (4695; 1:1000; Cell Signaling), phosphorylated ERK (4370; 1:2000; Cell Signaling), NF1 (sc-67; 1:50; Santa Cruz Biotechnology), Actin (A5316 or A5441; 1:5000; Sigma), or Vinculin (1:10000; ab1290002; Abcam). Bound primary antibodies were incubated for two hours at room temperature with IRDye800 conjugated affinity purified anti-rabbit or anti-mouse secondary antibodies (1:5000; Rockland, Gilbertsville, PA), and the signal was visualized using an Odyssey infrared imaging system (Li-Cor). Immunoblot band densities were determined with ImageJ (v1.46r, NIH) as previously described [39]. Relative intensity levels were determined by dividing the band intensity of the total protein by the intensity of the loading control protein and by dividing the intensity of the phosphorylated protein by the intensity of the total protein.

### Cell viability assays

The viability of cells exposed to binimetinib was determined using a modified methyl tetrazolium (MTT; Sigma) assay as previously described [39]. 0.35–0.9 × 10^5^ cells/ml of exponentially growing cells were plated in wells of 96-well plates. 24 h later, binimetinib was added to each well at specified concentrations, and the plates were incubated at 37 °C. 24, 48, 72, 96, or 120 h later, MTT was added to each well and plates were incubated at 37 °C for four h to allow for reduction of MTT to its insoluble formazan by remaining viable cells. Medium was aspirated and 150 μl of DMSO was added to each well to solubilize precipitated MTT. The optical density (OD) was immediately measured at 550 nm using a microplate spectrophotometer (Molecular Devices, Sunnyvale, CA). Relative cell viability was calculated by subtracting the background OD of media alone and then dividing by the OD of control wells. Replicates of six wells were used for each drug concentration and assays were duplicated on separate days. IC_50_ values were derived using best-fit trendlines as previously described [39].

To determine cell appearance before and after treatment with binimetinib, cells were plated as above and treated with either 1 μM or 10 μM binimetinib for 72 h. Cells were visualized using an inverted microscope (Nikon Eclipse TE-300, Nikon, Tokyo, Japan) and images were acquired on an RS Photometrics CoolSNAP color digital camera (Roper Scientific) using RS Photometrics Image Software Version 1.9.2 (Roper Scientific).

### Apoptosis assays

For assays to measure induction of apoptosis, 2 × 10^6^ neuroblastoma cells were plated in 60-mm plates and allowed to adhere and proliferate for 24 h. Cells were then treated with either 1 μM binimetinib, 10 μM binimetinib, or media alone (vehicle treatment) for six or eight hours (CHP-212 cells), 96 or 120 h (SJ-NB-10 cells), or 120 h only (CHP-134, NGP cells). Cells were harvested and lysed at the completion of each experiment as described above. Thirty please use mg (with symbol for "micro") total denatured protein from each cell line was separated by SDS-PAGE and transferred to nitrocellulose membranes (Invitrogen) as above. Western blots were performed as described above using primary antibodies to Poly(ADP-ribose) polymerase (PARP; 1:500, 9542, Cell Signaling) or Vinculin (1:10000; ab1290002; Abcam), anti-rabbit secondary antibody (1:5000; Rockland, Gilbertsville, PA), and the Odyssey infrared imaging system (Li-Cor).

### Analysis of patient outcomes compared to *NF1* and *MEK1* expression

We obtained microarray analysis results of neuroblastoma patient tumor samples from the National Cancer Institute (NCI) Oncogenomics Data Center Section (available at: http://pob.abcc.ncicrf.gov/cgi-bin/JK) from the databases “Neuroblastoma Prognosis Database,” “Neuroblastoma Prognosis Database-Oberthuer Lab,” and “Exon Array Neuroblastoma Database” as previously described [40]. All available patient data from these databases was included in our analysis. Using gene expression results from these databases, patients were divided into high and low *NF1* and *MEK1* gene expression groups by median-centered log2 ratios as detailed on the NCI Oncogenomics database website. Kaplan-Meier survival curves were plotted using the open-source statistical packages in R (R Foundation for Statistical Computing, Vienna, Austria; available at: http://www.r-project.org). We compared survival curves between the *NF1* and *MEK1* gene expression groups using log-rank tests to examine the association between expression and patient survival outcomes in the whole cohort and in patients with stage 4 neuroblastoma and in those with stage 1, 2, 3, or 4S neuroblastoma.

We obtained additional microarray analysis results of neuroblastoma patient tumor samples from the R2 Genomics Analysis and Visualization Platform (http://r2.amc.nl) using the Versteeg database. *MEK1 and MEK2* probesets in each database with the highest average signals were selected for analysis. Kaplan-Meier analyses were performed online and the resulting survival curves and p values (obtained via the log-rank test) were downloaded as previously described [41].

## Results

### Neuroblastoma patient samples and tumor cell lines demonstrate RAS/MAPK pathway expression and activity

**Fig. 1:**
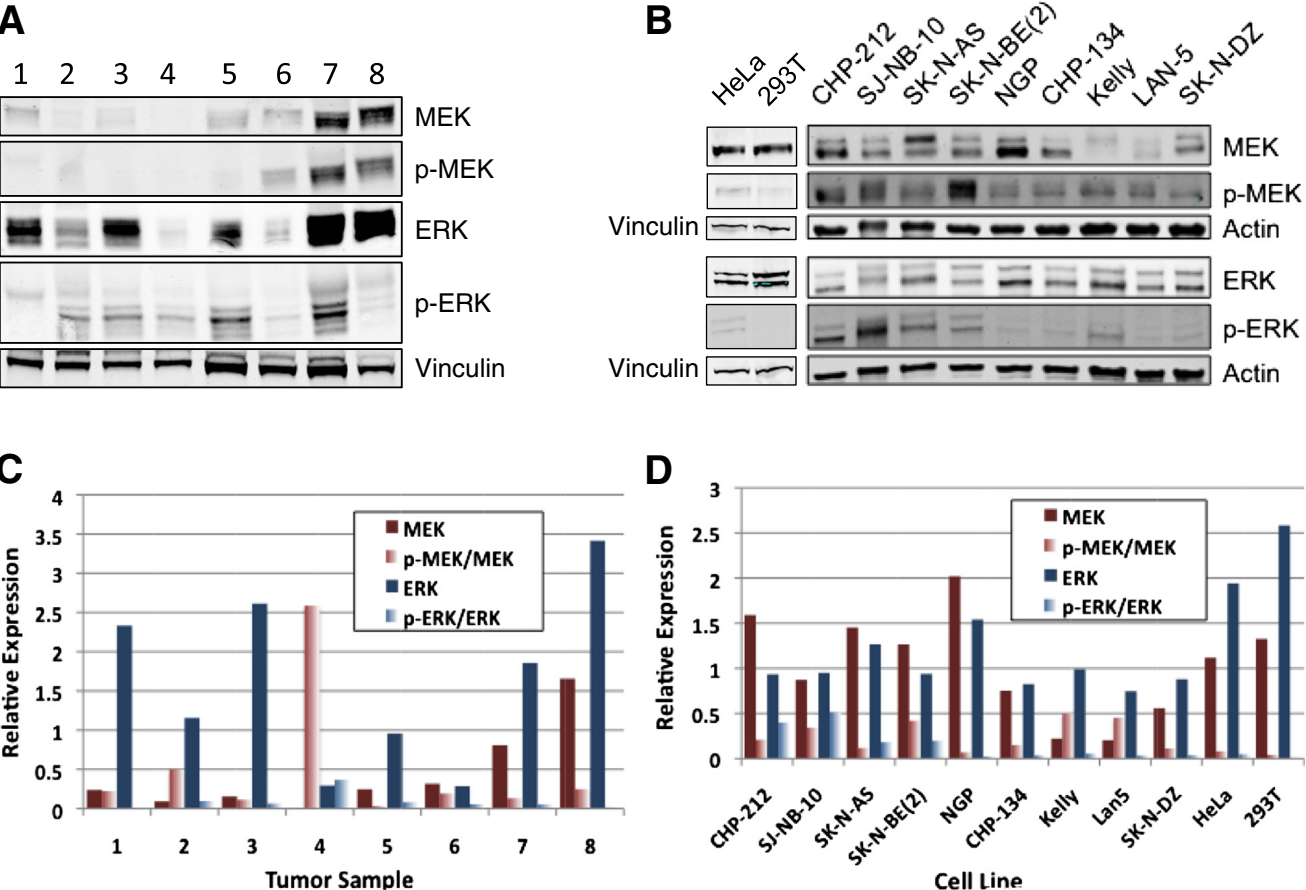
Neuroblastoma patient samples and cell lines show expression and activity of components of the RAS/MAPK signaling pathway. **a** Neuroblastoma patient samples were lysed and Western blots for total MEK, phosphorylated MEK (p-MEK), total ERK, and phosphorylated ERK (p-ERK) were performed. Vinculin was used as a loading control. **b** A panel of nine neuroblastoma cell lines, HeLa cells and 293T cells were lysed and Western blots for total MEK, p-MEK, total ERK, and p-ERK were performed. Actin and vinculin were used as loading controls. **c**, **d** Relative MEK, p-MEK, ERK, and p-ERK western blot band intensities were determined and plotted for each tested tumor sample and cell line

To examine the expression of components of the RAS/MAPK signaling pathway in neuroblastoma tumors, a cohort of patient tumor samples was analyzed by Western blot for total and phosphorylated MEK and ERK. Patient tumor samples showed a range of expression of total and phosphorylated components of this pathway (Fig. 1a, c). Neuroblastoma cell lines also showed varying levels of total and phosphorylated MEK and ERK (Fig. 1b, d). Although there was no apparent correlation between levels of phosphorylated MEK and phosphorylated ERK in these samples and cell lines, detectable levels of both phosphorylated MEK and ERK suggested activity of this pathway in neuroblastoma tumor cells and also suggested the potential efficacy of MEK inhibitors in neuroblastoma preclinical models.

### Neuroblastoma tumor cell responses to binimetinib

**Fig. 2:**
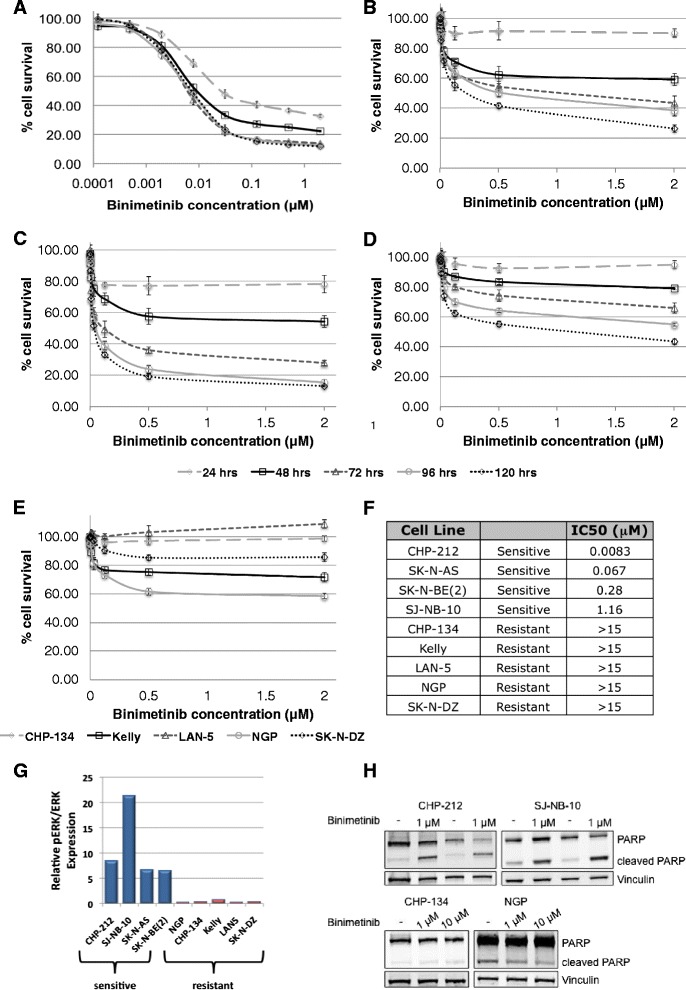
Neuroblastoma cell lines show bimodal responses to treatment with the MEK1/2 inhibitor binimetinib. **a-f** Neuroblastoma cells were treated with increasing concentrations of binimetinib for 24, 48, 72, 96, or 120 h and cell viability was determined by MTT assays. CHP-212 (log scale) (**a**), SK-N-BE(2) (**b**), SK-N-AS (**c**), and SJ-NB-10 (**d**) cells are sensitive to binimetinib treatment; **e** CHP-134, Kelly, LAN-5, NGP, and SK-N-DZ cells maintain resistance to binimetinib treatment after 120 h of drug exposure. **f** IC_50_ values (μM) were calculated for cells treated with binimetinib for 120 h. **g** Densitometry analysis was performed on Western blots from Fig. 1b to quantify relative phospho-ERK (pERK/ERK) protein levels in neuroblastoma tumor cell lines sensitive to binimetinib (“sensitive”) or resistant to binimetinib (“resistant”) **h** CHP-212 cells were treated with 1 μM binimetinib for 6 h (left two lanes) or 8 h (right two lanes) and SJ-NB-10 cells were treated with 1 μM binimetinib for 96 h (left two lanes) or 120 h (right two lanes). CHP-134 and NGP cells were treated with 1 μM or 10 μM binimetinib for 120 h. Cells were then lysed and Western blots for total and cleaved PARP were performed. Vinculin was used as a loading control

With the demonstrated activity of the RAS/MAPK pathway in neuroblastoma tumor cells and tumors, we hypothesized that MEK inhibition would lead to decreased cell viability. To investigate this hypothesis, neuroblastoma tumor cell lines were tested for sensitivity *in vitro* to the novel MEK1/2 inhibitor binimetinib. Four cell lines were sensitive to binimetinib and reached <50 % viability after 24 to 120 h of treatment (Fig. 2a–d) while five cell lines were resistant to the drug (Fig. 2e). Resistant cell lines were largely unaffected by treatment with binimetinib for up to five days with doses up to 15 μM (Fig. 2e, Additional file 1), while IC_50_ values for the sensitive cell lines ranged from 8 nM to 1.16 μM after 120 h of drug treatment (Fig. 2f). Resistant cell lines did not demonstrate any significant morphological changes in response to binimetinib, while sensitive cell lines demonstrated cell rounding and detachment from the surface, consistent with cell death (Additional file 2). Responsiveness of cells to binimetinib correlated with their levels of RAS/MAPK signaling pathway activity. Cell lines more sensitive to binimetinib tended to show higher levels of phosphorylated MEK and ERK proteins (Fig. 2g), while cell lines least sensitive to binimetinib showed lower levels of phosphorylated MEK and ERK proteins (Fig. 2g).

In order to determine the mechanism of decreased neuroblastoma tumor cell viability after treatment with binimetinib, we analyzed cells for cleavage of PARP before and after treatment with binimetinib. Treatment with binimetinib led to an increase in PARP cleavage in sensitive but not resistant cell lines (Fig. 2h), indicating that the reduction in viability from binimetinib treatment is at least partially due to induction of apoptosis in sensitive cell lines.

### Binimetinib inhibits RAS/MAPK pathway activity

**Fig. 3:**
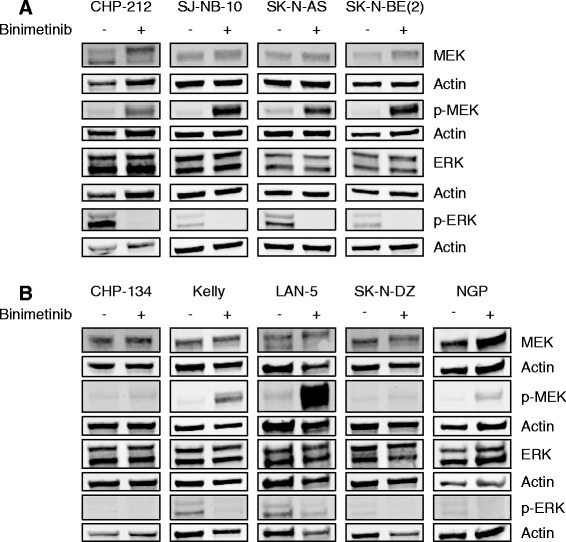
Binimetinib inhibits RAS/MAPK pathway activity. Neuroblastoma cells were treated with 1 μM binimetinib for 1 h and then lysed and Western blots for total MEK, phospho-MEK (p-MEK), total ERK, and phospho-ERK (p-ERK) were performed. Actin was used as a loading control

In order to demonstrate inhibition of MEK and ERK in neuroblastoma tumor cells, neuroblastoma tumor cell lines were treated with binimetinib or media alone for 1 h. Treatment of sensitive cell lines with binimetinib led to increased MEK phosphorylation and inhibition of ERK phosphorylation without changes in total levels of MEK and ERK protein (Fig. 3a, b). Resistant cell lines demonstrated either less robust increases in phosphorylation of MEK or incomplete inhibition of phosphorylated ERK (Fig. 3b), suggesting multiple possible mechanisms of resistance.

### NF1 expression correlates with responses of cells to binimetinib

**Fig. 4:**
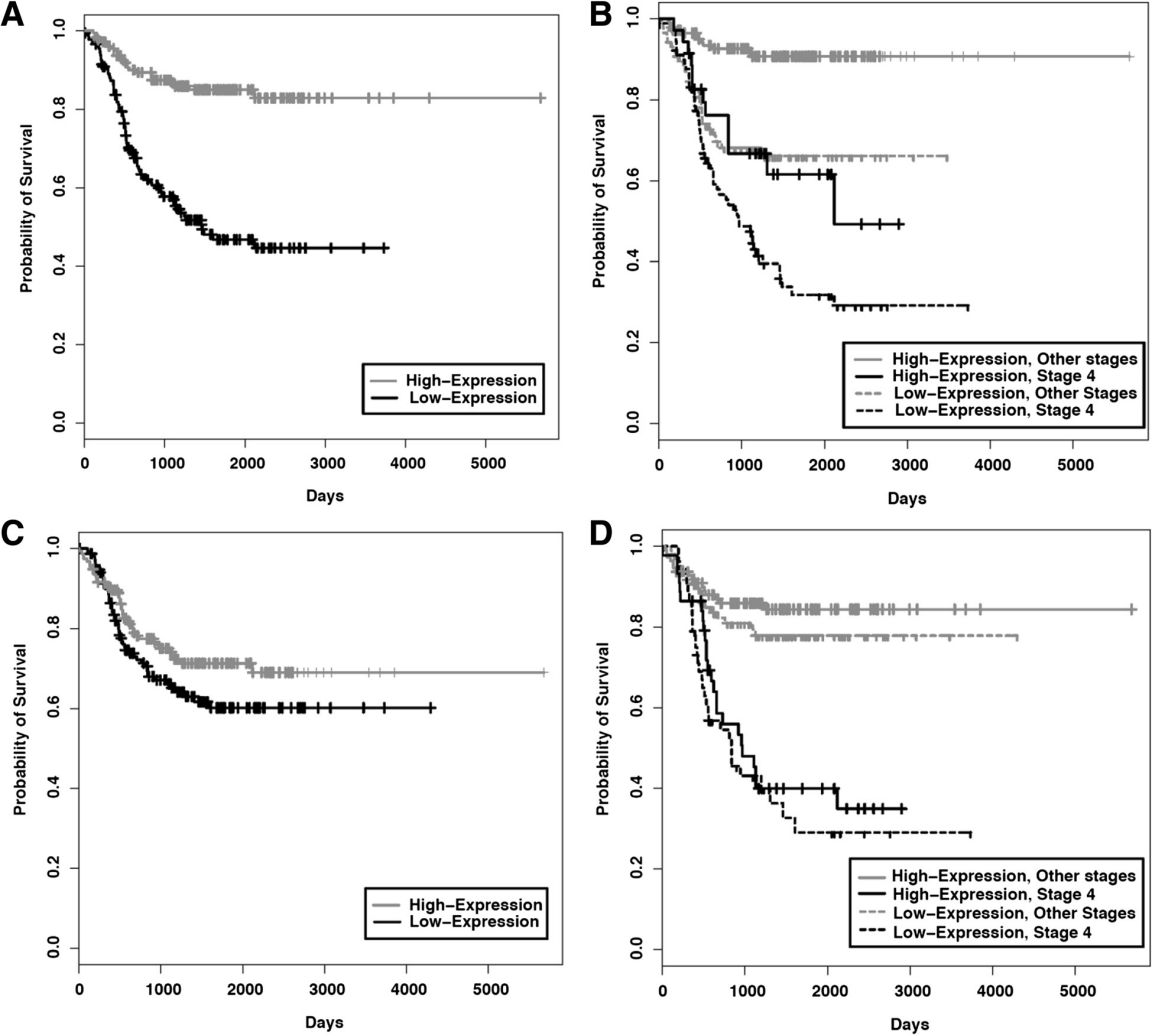
Outcomes of patients with neuroblastoma based on *MEK1* and *NF1* gene expression. The NCI Oncogenomics gene expression databases were evaluated for outcomes of patients with neuroblastoma and Kaplan-Meier survival curves were generated. **a** Estimated overall survival for patients who have tumors with high *NF1* gene expression (*n* = 177; gray) and low *NF1* gene expression (*n* = 176; black) (log-rank test; *p* = 1.88e-11). **b** Estimated overall survival for patients with stage 4 neuroblastoma who have high (*n* = 35; black) and low (*n* = 90; dashed black) *NF1* gene expression and for patients with tumors of all other stages with high (*n* = 142; gray) and low (*n* = 86; dashed gray) *NF1* gene expression. **c** Estimated overall survival for patients who have tumors with high *MEK1* gene expression (*n* = 154; gray) and low *MEK1* gene expression (*n* = 153; black) (log-rank test; *p* = 0.44). **d** Estimated overall survival for patients with stage 4 neuroblastoma who have high (*n* = 44; black) and low (*n* = 54; dashed black) *MEK1* gene expression and for patients with tumors of all other stages with high (*n* = 110; gray) and low (*n* = 99; dashed gray) *MEK1* gene expression

Expression of the RAS-GTPase activating protein (GAP) protein NF1 is associated with activity of the RAS/MAPK pathway, and mutations in or deletions of the *NF1* gene have been found in a number of cancers, including neuroblastoma [29, 42]. To evaluate whether gene expression of RAS/MAPK pathway members was associated with neuroblastoma patient outcomes, we evaluated the associations of *NF1* and *MEK1/2* gene expression with neuroblastoma patient outcomes using results from microarray analyses of neuroblastoma tumors. *NF1* gene expression, but not *MEK1 or MEK2* gene expression, was strongly associated with patient outcomes in neuroblastoma and appeared to have prognostic effects independent of tumor stage (Fig. 4; Additional file 3).

**Fig. 5:**
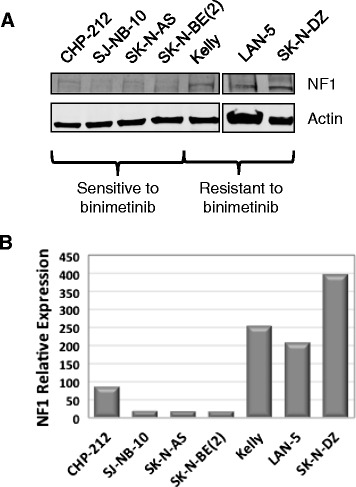
Neuroblastoma cell lines sensitive to binimetinib show lower levels of NF1 protein expression. **a** A panel of neuroblastoma cell lines was analyzed by Western blot for NF1 protein expression levels. Actin was used as a loading control. **b** Densitometry analysis was performed to quantify relative NF1 protein levels in neuroblastoma tumor cell lines sensitive or resistant to binimetinib

Loss of or reduced expression of *NF1* leads to hyperactivation of RAS and of its downstream signaling components such as MEK and ERK [43–45]. Thus, we hypothesized that NF1 expression in neuroblastoma tumor cells might influence responses to binimetinib treatment. To determine whether NF1 protein levels correlated with responses to binimetinib, we examined levels of NF1 protein in neuroblastoma cell lines. Cell lines sensitive to binimetinib treatment had the lowest NF1 protein levels, while resistant cell lines showed the highest levels of NF1 protein (Fig. 5), suggesting that NF1 levels may be useful as a biomarker to identify neuroblastoma patients that would be more likely to respond to MEK inhibitor therapy.

## Discussion

New treatment strategies are sorely needed for patients with high risk and relapsed neuroblastoma, and we have shown that MEK inhibition with binimetinib may represent an effective therapy for these patients. We have shown that neuroblastoma tumor cells and patient samples show expression and activity of components of the RAS/MAPK signaling pathway, supporting a role for this pathway in neuroblastoma pathogenesis. We have also shown that multiple neuroblastoma tumor cell lines were sensitive to treatment with the MEK inhibitor binimetinib, with sensitivity to MEK inhibition linked to NF1 protein expression and levels of phosphorylated MEK and ERK. Furthermore, we have shown that *NF1* gene expression is associated with neuroblastoma patient outcomes, suggesting that MEK inhibitors would be most effective in patients with the worst outcomes and that NF1 expression represents a potentially useful biomarker for response to RAS/MAPK pathway inhibition.

GTPase-activating proteins (GAPs), including NF1, function as negative regulators of RAS. RAS cycles between an active, GTP-bound state and an inactive, GDP-bound conformation. The interaction between RAS and NF1 accelerates the conversion of RAS-GTP to RAS-GDP, therefore downregulating the activity of RAS, and loss of *NF1* leads to hyperactivation of RAS and of its downstream signaling components such as MEK and ERK [43–45]. Previous work has identified *NF1* gene deletions in multiple neuroblastoma cell lines [29], likely contributing to the lack of NF1 protein seen in these cell lines and suggesting that neuroblastoma tumors with reduced or absent NF1 expression are likely to be sensitive to MEK inhibition.

Upon treatment with binimetinib, neuroblastoma tumor cells show a bimodal response with some cells being very sensitive and others being resistant. Our data indicates that binimetinib strongly suppresses ERK activity in the sensitive cell lines, leading to apoptosis. However, multiple neuroblastoma tumor cells are resistant to inhibition of MEK with binimetinib, with either incomplete inhibition of ERK phosphorylation or reduced increases in MEK phosphorylation after treatment. Therefore, mechanisms of resistance could include alternative signaling pathways or feedback loops activating ERK in the absence of MEK activity, leading to resistance to binimetinib. Research is ongoing to identify these pathways mediating resistance to MEK inhibitor therapy.

Binimetinib treatment in adult cancer patients was generally well-tolerated but was associated with mild to moderate central serous-like retinopathy, diarrhea and acneiform dermatitis, similar to other MEK inhibitors [25, 26, 46]. Currently, multiple clinical trials examining the safety and efficacy of binimetinib alone and in conjunction with other drugs for cancer therapy are ongoing. MEK inhibition with binimetinib also results in the inhibition of other normal physiologic processes, such as inflammation [16, 47]. Therefore, it will be crucial to identify patient subpopulations most likely to benefit from MEK inhibitor therapy to minimize the risk:benefit ratio for patients.

## Conclusions

Currently, more than a dozen inhibitors of MEK1 and MEK2 are in clinical development, including binimetinib. In clinical trials, such inhibitors have shown a range of efficacy. Unfortunately, in many cases, patients fail to initially respond to treatment; in other cases, patients respond well at the onset of treatment but later develop mechanisms of resistance to such drugs. Being able to identify an appropriate patient population that will respond to inhibition of MEK would facilitate the effective development and use of such inhibitors. Our data has supported a model in which levels of NF1 and phosphorylated MEK and ERK influence the sensitivity of cell lines to MEK inhibition with binimetinib. However, levels of phosphorylated MEK and ERK would be difficult both to obtain and quantify, and therefore our data identifying NF1 as a biomarker that predicts both patient outcomes and responsiveness of neuroblastoma tumor cells to MEK inhibition supports a potential role for readily available genetic testing for *NF1* mutations and deletions in tumor samples. Thus, NF1 may not only function as an easily obtainable prognostic marker to predict disease outcomes but NF1 gene and protein expression levels may also represent independent molecular markers for a subset of neuroblastoma patients that is in need of additional therapies and that may respond well to MEK inhibition.

## Additional files

Additional file 1: CHP-134, Kelly, LAN-5, NGP, and SK-N-DZ cells remain resistant to binimetinib at doses exceeding 15 μM. Neuroblastoma cells were treated with increasing concentrations of binimetinib for 120 h and cell viability was determined by MTT assays. (PPTX 60 kb) Additional file 2: Effects of binimetinib on neuroblastoma tumor cell morphology. Neuroblastoma tumor cells were photographed before treatment and after treatment with 1 μM or 10 μM binimetinib for 72 h. (PPTX 12467 kb) Additional file 3: Using the neuroblastoma Versteeg patient data-sets in the R2 Genomics Analysis and Visualization Platform (http://r2.amc.nl), patients were divided into high (blue) and low (red) *MEK1 (left) and MEK2 (right)* gene expression groups by median-centered Log2 ratios and survival curves were generated. Overall survival curves are shown with patient numbers in parentheses. (PPTX 144 kb)

## Abbreviations

ATCC: American Type Culture Collection
DMSO: dimethyl sulfoxide
ERK: extracellular signal-regulated kinase
FBS: fetal bovine serum
MAPK: mitogen-activated protein kinase
MEK: MAPK/ERK kinase
MTT: 3-(4,5 dimethylthiazolyl-2-yl)-2,5-diphenyltetrazolium bromide
NF1: neurofibromatosis type 1
PARP: poly(ADP-ribose) polymerase
PVDF: polyvinylidene fluoride
RIPA: radioimmunoprecipitation assay
SDS-PAGE: sodium dodecyl sulfate-polyacrylamide gel electrophoresis
