# Supplementary figures and images for: Binimetinib inhibits MEK and is effective against neuroblastoma tumor cells with low NF1 expression
Source: BMC Cancer. 2016 Mar 1;16:172. doi: 10.1186/s12885-016-2199-z (PMC4772351; doi:10.1186/s12885-016-2199-z)

## Slide 1
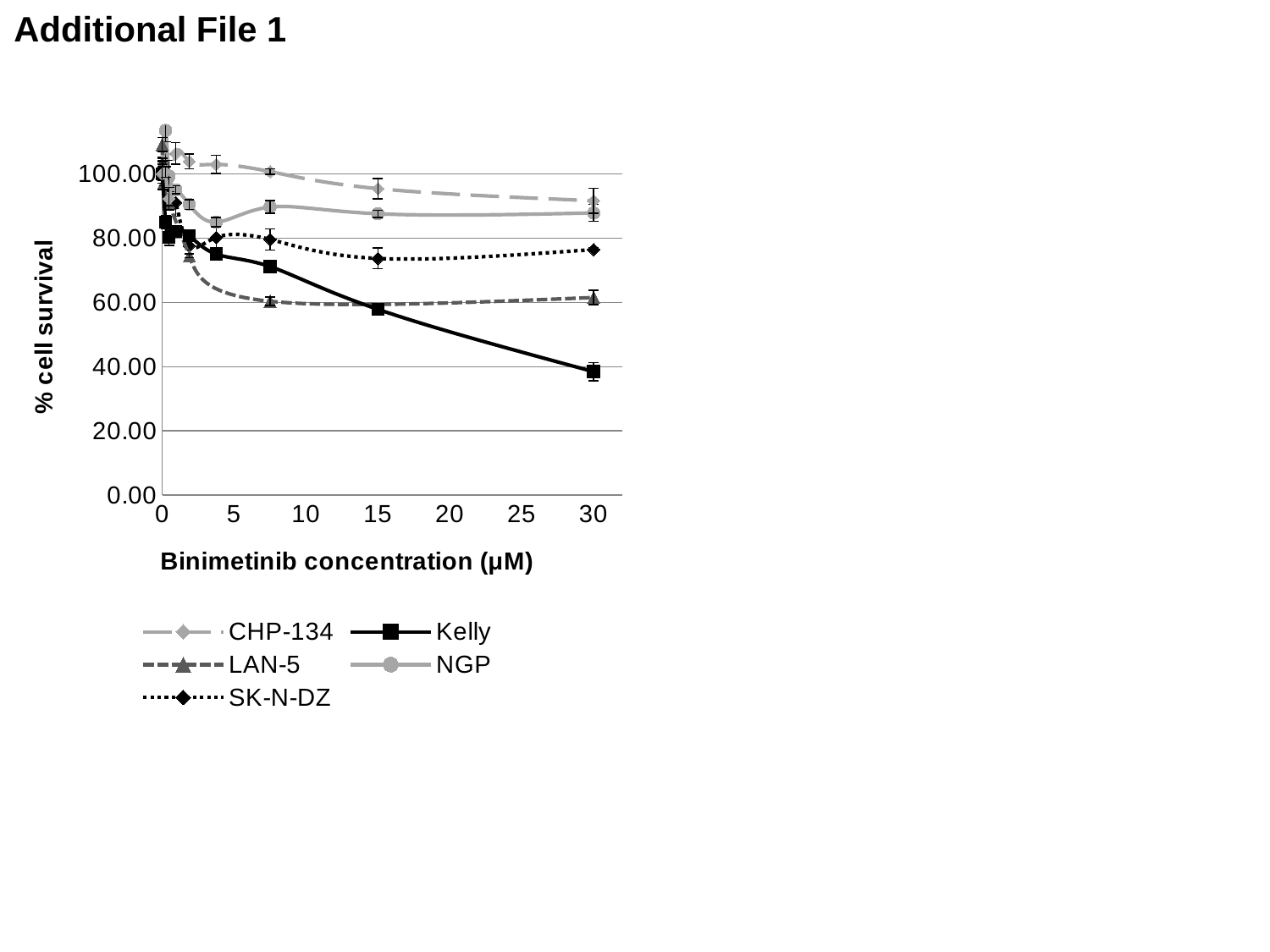

Additional File 1
### Chart
| Category | | | | | |
|---|---|---|---|---|---|

Supplement: Additional file 1: — CHP-134, Kelly, LAN-5, NGP, and SK-N-DZ cells remain resistant to binimetinib at doses exceeding 15 μM. Neuroblastoma cells were treated with increasing concentrations of binimetinib for 120 h and cell viability was determined by MTT assays. (PPTX 60 kb) [file 12885_2016_2199_MOESM1_ESM.pptx]

## Slide 1
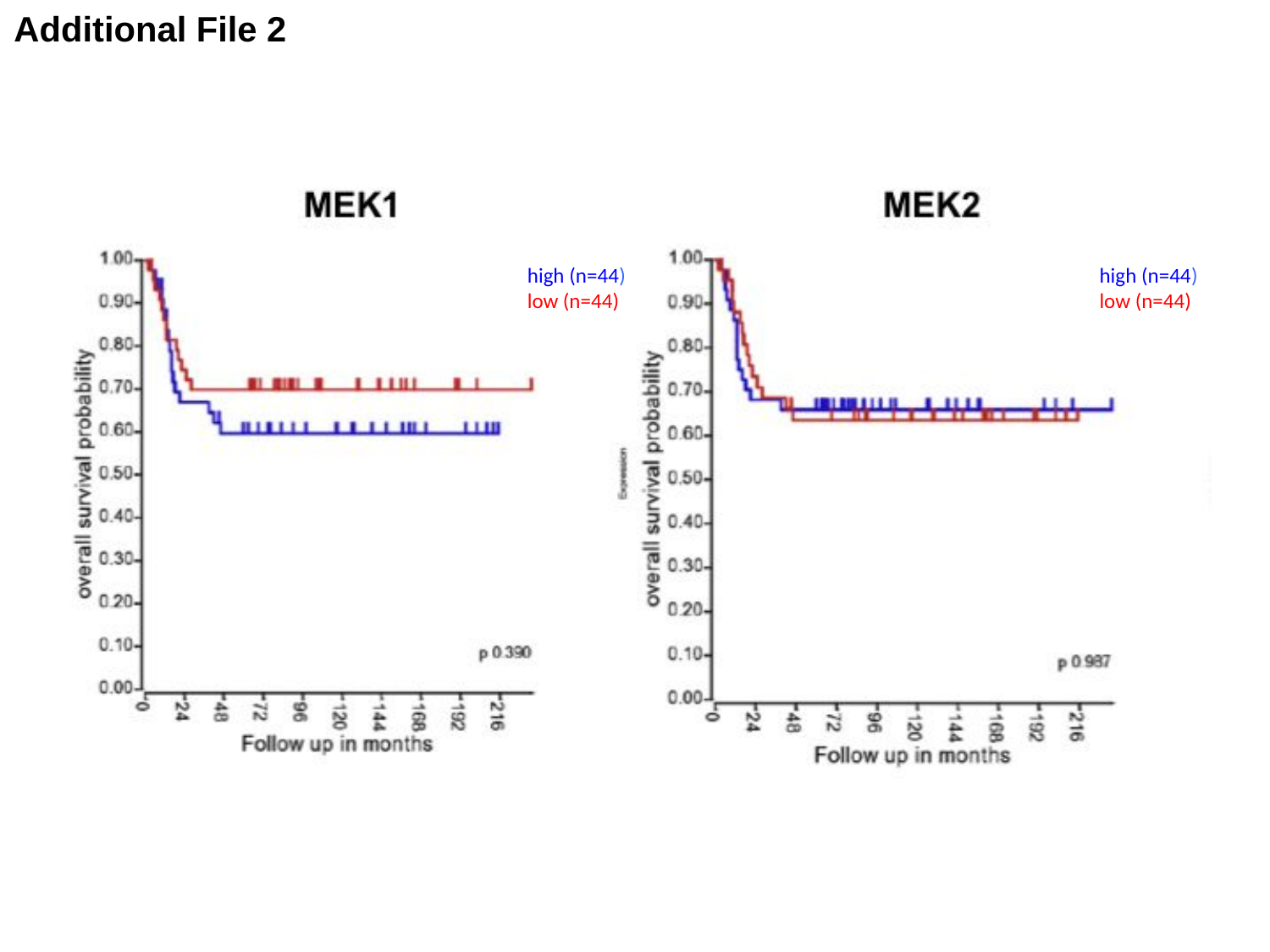

Additional File 2
high (n=44)
low (n=44)
high (n=44)
low (n=44)

Supplement: Additional file 3: — Using the neuroblastoma Versteeg patient data-sets in the R2 Genomics Analysis and Visualization Platform (http://r2.amc.nl), patients were divided into high (blue) and low (red) MEK1 (left) and MEK2 (right) gene expression groups by median-centered Log2 ratios and survival curves were generated. Overall survival curves are shown with patient numbers in parentheses. (PPTX 144 kb) [file 12885_2016_2199_MOESM3_ESM.pptx]
