# Supplementary material for: Binimetinib inhibits MEK and is effective against neuroblastoma tumor cells with low NF1 expression
Source: BMC Cancer. 2016 Mar 1;16:172. doi: 10.1186/s12885-016-2199-z (PMC4772351; doi:10.1186/s12885-016-2199-z)
Supplement: Additional file 2: — Effects of binimetinib on neuroblastoma tumor cell morphology. Neuroblastoma tumor cells were photographed before treatment and after treatment with 1 μM or 10 μM binimetinib for 72 h. (PPTX 12467 kb) [file 12885_2016_2199_MOESM2_ESM.pptx]

## Slide 1
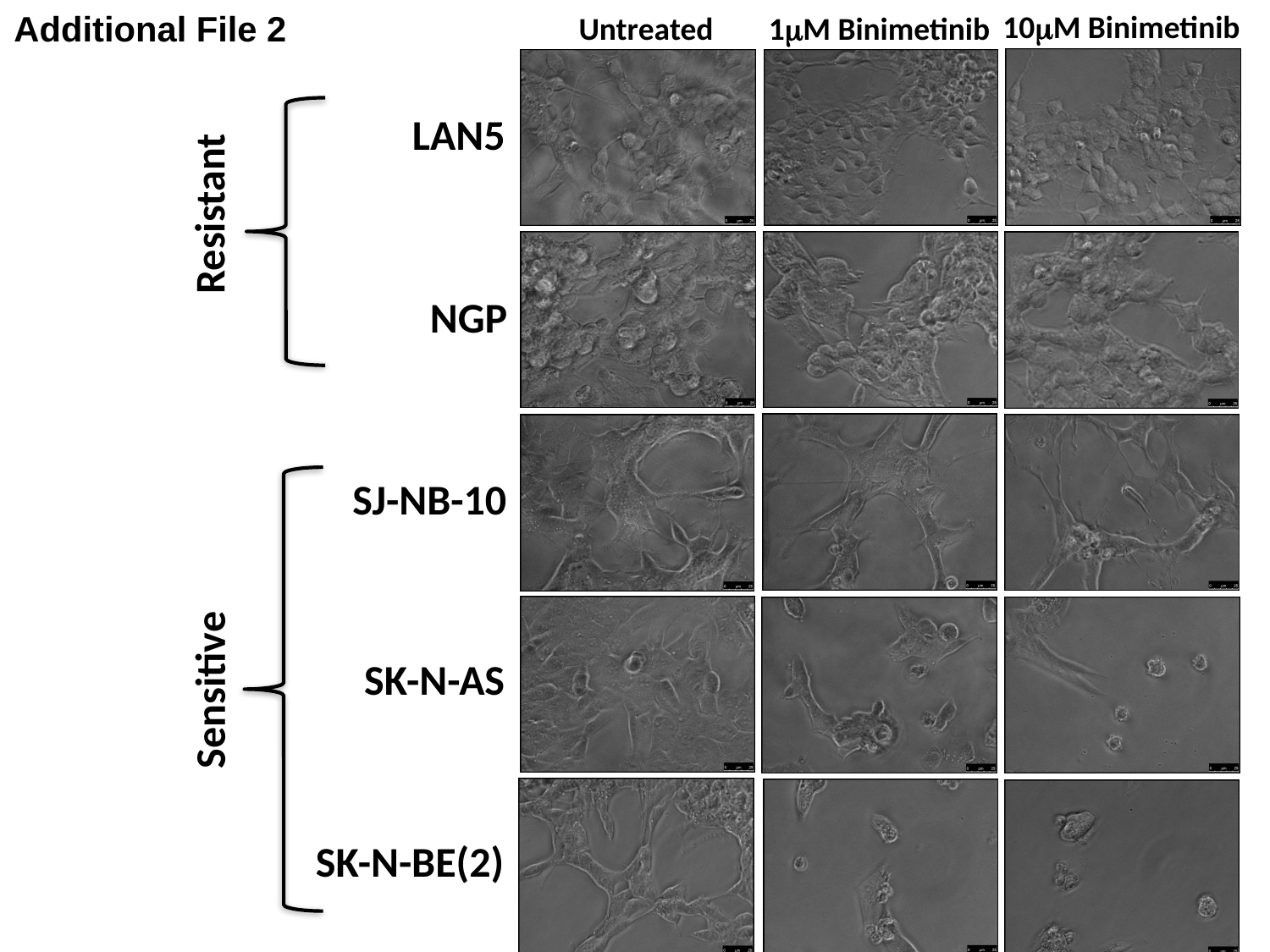

Additional File 2
10mM Binimetinib
1mM Binimetinib
Untreated
LAN5
Resistant
NGP
SJ-NB-10
SK-N-AS
Sensitive
SK-N-BE(2)
